# Association study between SNP rs150689919 in the DNA demethylation gene, *TET1*, and Parkinson’s disease in Chinese Han population

**DOI:** 10.1186/1471-2377-13-196

**Published:** 2013-12-11

**Authors:** Xin-xin Liao, Zi-xiong Zhan, Ying-ying Luo, Kai Li, Jun-ling Wang, Ji-feng Guo, Xin-xiang Yan, Kun Xia, Bei-sha Tang, Lu Shen

**Affiliations:** 1 Department of Neurology, Xiangya Hospital, Central South University, Changsha, Hunan 410008, P. R. China; 2 State Key Laboratory of Medical Genetics, Central South University, Changsha, Hunan 410078, P. R. China; 3 Key Laboratory of Neurodegenerative Disorders in Hunan Province, Central South University, Changsha, Hunan 410008, P. R. China

**Keywords:** *TET1*, Parkinson’s disease, Epigenetics, Chinese

## Abstract

**Background:**

Recent studies suggest that epigenetic factors may play an important role in the pathogenesis of Parkinson’s disease (PD). In our previous work, we sequenced the exomes of sixteen patients from eight Chinese PD families using whole exome sequencing technology, consequently three patients from different pedigrees were found sharing the variant c.1460C > T (rs150689919) in the coding region of the Tet methyl cytosine dioxygenase 1 (*TET1*) gene.

**Methods:**

In order to evaluate the possible association between sporadic PD and the single nucleotide polymorphism (SNP) rs150689919 in *TET1*, a case–control cohort study was conducted in 514 sporadic PD patients and 529 normal controls. Genotyping was determined by PCR and direct sequencing. Statistical significance was analyzed by the Chi-squared test.

**Results:**

There was no statistical significance in *TET1* rs150689919 genotype or allele frequencies between the PD cases and healthy controls, even after being stratified by gender and age at onset.

**Conclusions:**

Our findings suggest that rs150689919 in *TET1* may not be associated with PD in Chinese population. However, due to the limited data in this study, replication studies in larger sample and other populations are required.

## Background

Parkinson’s disease (PD) is the second most common progressive neurodegenerative disorder worldwide after only Alzheimer’s disease. Resting tremors, muscular rigidity, bradykinesia, postural instability, and positive response to dopamine replacement therapy are the main clinical manifestations of the condition [1]. Familial forms represent only a minority of the cases (ranging from 5 to 10% of the total), whereas the vast majority of PD occurs as sporadic forms [2]. Although the etiology of idiopathic PD remains unclear, there is a growing body of evidence suggesting that a large proportion of these cases are also significantly influenced by genetic factors. Genetic association studies based on the “candidate gene approach” and genome-wide association studies have revealed several genetic variants that might act as susceptibility factors for the sporadic cases [http://www.pdgene.org/] [3].

Recently, exome sequencing has emerged as a feasible, cost-effective, and high-throughput strategy that enables the detection of rare coding variants, offering new insight to investigate the association between rare variants and complex diseases. In our previous work, whole exome capture and high-throughput sequencing technology were carried out in 16 patients from eight pedigrees clinically diagnosed as PD to identify the potential causative genes (data not shown). Many studies have shown that some causative genes for Mendelian-inherited PD, such as *SNCA* and *PARK16*, were also identified and confirmed as susceptibility genes for sporadic PD [http://www.pdgene.org/] [3]. Therefore, we performed a comparison of the exome data to screen for some potential susceptibility variants for idiopathic PD. Because rare variants might have more marked functional consequences, we first compared the non-synonymous variants against dbSNP135, eight previous exome-sequenced HapMap samples (HapMap 8), and the single nucleotide polymorphism (SNP) release of the 1000 Genome Project (20100208 release). The shared SNPs that had the population minor allele frequencies (MAFs) more than 5% were removed. Subsequently, prediction tools SIFT [http://sift.jcvi.org/] and PolyPhen-2 [http://genetics.bwh.harvard.edu/pph2/index.shtml] were used to assess the non-synonymous variants and identify those likely to have a functional impact [4]. The variants that affected the highly conserved sequences or that were predicted to be deleterious were selected. As a result, 28 variants from 25 genes had higher frequencies in sixteen patients were found (see Additional file 1: Table S1).

In order to identify the most promising candidate variant, the functional analysis of the 25 genes were conducted, and the DNA demethylation gene, *TET1*, encodes the human ten-eleven translocation 1 protein, was brought to attention. Firstly, the variant rs150689919 in *TET1* had the highest frequency in the sixteen patients (three patients from different families shared this variant, see Additional file 2: Figure S1). Secondly, TET1 protein can convert 5-methylcytosine (5mC) into 5-hydroxymethylcytosine (5hmC), 5-formylcytosine (5fC), and 5-carboxylcytosine (5caC) through three consecutive oxidation reactions [5,6], and these modified bases may represent new epigenetic states in genomic DNA or intermediate in the process of DNA demethylation [7,8]. As we know, substantial evidences already revealed that methylation dysfunction and expression changes were existed in PD and some PD risk variants in *SNCA*, *PARK16*, *GPNMB* and *STX1B*[9,10]. Considering the dysfunction of epigenetic machinery may play an important role in the pathogenesis of PD [10,11], and the relatively high frequency in our familial PD cases, we chose rs150689919 in *TET1* as the candidate variant to perform a case–control cohort study to further investigate the possible association between the SNP and sporadic PD.

## Methods

### Subjects

A total of 514 ethnic Han Chinese PD patients (267 males, 247 females) from the Department of Neurology, Xiangya Hospital and the Key Laboratory of Neurodegenerative Disorders in Hunan Province were enrolled in this study. The patients had a mean age at onset of 54.82 ± 12.19 years (range: 11-91 years). Early onset Parkinson’s disease (EOPD) was defined as PD with an age at onset less than 50 years (166 subjects), with the mean age at onset of 40.89 ± 8.46 years (range: 11-50 years). Late Onset Parkinson’s disease (LOPD) was defined as PD with an age at onset more than 50 years (348 subjects), with the mean age at onset of 61.47 ± 6.97 years (range: 51-91 years). All of the subjects were evaluated by two experienced neurologists and diagnosed as idiopathic PD based on the United Kingdom Parkinson’s Disease Society Brain Bank Clinical Diagnostic Criteria [12]. None of the patients had a reported family history of PD in one or more first-or second-degree relatives. In addition, 529 unrelated individuals without symptoms of Parkinsonism, Alzheimer’s disease, other extrapyramidal diseases as well as family history of parkinsonism were enrolled in the study and matched for age, gender, ethnicity, and area of residence (261 males and 268 females; mean age 53.55 ± 6.26 years; range 15-90 years). The study was approved by the Ethics Committee of Xiangya Hospital, Central South University. A written informed consent was obtained from each subject involved in the research.

### Polymorphism analysis

Blood samples were collected from all subjects using ethylenediaminetetraacetic acid (EDTA) tubes. Genomic DNA was extracted from peripheral blood leukocytes using the standard phenol-chloroform extraction method. The genotype was detected by direct sequencing using an ABI 3100 automated sequencer (Applied Bio systems, Foster City, CA). A 637-bp fragment containing the variant was amplified using the following primers: forward 5′-AGTTTCTGATACCACCTCTTTCC-3′, and reverse 5′-GACCATTGGCACTGGCATAG-3′. Polymerase chain reaction (PCR) was performed using the following amplification conditions: 32 cycles of denaturation at 94°C for 45 s, annealing at 64.6°C for 45 s, and extension at 72°C for 45 s. A final extension was performed for 10 min at 72°C.

### Statistical analysis

The Chi-squared test was used to test for allele and genotype frequencies of the PD patients and controls. A p-value < 0.05 using a two-tailed test was considered statistically significant. Statistical analysis was carried out using SPSS 17.0 software (SPSS Inc., Chicago, IL, USA).

## Results

Genotype and allele frequencies for all 1058 subjects (514 patients and 529 healthy controls) were shown in Table 1. The SNP was in agreement with Hardy-Weinberg equilibrium in both groups. There was no significant difference in genotype and allele frequencies between cases and controls (p = 0.137 for genotype frequency; p = 0.361 for allele frequency). Genotype and allele frequencies of rs150689919 in patients and controls of both genders were shown in Table 2. No statistically significant difference was found in either genotype or allele frequencies between male or female cases and controls (male, p = 0.208 and p = 0.261, respectively; female, p = 0.517 and p = 0.871, respectively). The genotype and allele frequencies of rs150689919 in EOPD and LOPD cases were shown in Table 3. There was no significant difference observed in either allele or genotype distribution in EOPD or LOPD cases compared to controls (EOPD, p = 0.296 and p = 0.176, respectively; LOPD, p = 0.671 and p = 0.176, respectively).

**Table 1:** Genotype and allele frequencies of SNP rs150689919 in PD patients and controls

**SNP**	**N**	**Genotype N (%)**			***p *** **(df, OR)**	**Allele N (%)**		***p *** **(df, OR)**
		**CC**	**CT**	**TT**		**C**	**T**	
Patients	514	474 (92.2)	38 (7.4)	2 (0.4)	0.137^a^ (df = 2, OR^*^)	986 (95.9)	42 (4.1)	0.361^b^ (df = 1,OR = 0.824)
Controls	529	477 (90.2)	52 (9.8)	0		1006 (95.1)	52 (4.9)	

*p*-Values after correcting by binary logistic regression with age and gender using SPSS17.0.

^a^*p* = 0.363; ^b^*p* = 0.362.

^*^With df = 2, the OR value could not been given by Chi-square test.

**Table 2:** Genotype and allele frequencies of rs150689919 in male/female PD patients and controls

**SNP**	**N**	**Genotype N (%)**			***p *** **(df, OR)**	**Allele N (%)**		***p *** **(df, OR)**
		**CC**	**CT**	**TT**		**C**	**T**	
Male	267	248 (92.9)	18 (6.7)	1 (0.4)	0.208^a^ (df = 2, OR*)	514 (96.3)	20 (3.7)	0.261^b^ (df = 1, OR = 0.713)
Controls	261	234 (89.7)	27 (10.3)	0		495 (94.8)	27 (5.2)	
Female	247	226 (91.5)	20 (8.1)	1 (0.4)	0.517^c^ (df = 2, OR^*^)	472 (95.5)	22 (4.5)	0.871^d^ (df = 1, OR = 0.953)
Controls	268	243 (90.7)	25 (9.3)	0		511 (95.3)	25 (4.7)	

*p*-Values after correcting by binary logistic regression with age using SPSS17.0.

^a^*p* = 0.267; ^b^*p* = 0.266; ^c^*p* = 0.881; ^d^*p* = 0.881.

^*^With df = 2, the OR value could not been given by Chi-square test.

**Table 3:** Genotype and allele frequencies of rs150689919 in EOPD/LOPD patients and controls

**SNP**	**N**	**Genotype N (%)**			***p *** **(df, OR)**	**Allele N (%)**		***p *** **(df, OR)**
		**CC**	**CT**	**TT**		**C**	**T**	
EOPD	166	156 (94.0)	9 (5.4)	1 (0.6)	0.176^a^ (df = 2, OR^*^)	321 (96.7)	11 (3.3)	0.296^b^ (df = 1, OR = 0.669)
Controls	195	176 (92.3)	19 (9.7)	0		371 (95.1)	19 (4.9)	
LOPD	348	318 (91.4)	29 (8.3)	1 (0.3)	0.487^c^ (df = 2, OR^*^)	665 (95.5)	31 (4.5)	0.671^d^ (df = 1, OR = 0.897)
Controls	334	301 (90.1)	33 (9.9)	0		635 (95.6)	33 (4.7)	

*p*-Values after correcting by binary logistic regression with age and gender using SPSS17.0.

^a^*p* = 0.423; ^b^*p* = 0.432; ^c^*p* = 0.665; ^d^*p* = 0.668.

EOPD, early onset Parkinson’s disease (age at onset ≤ 50); LOPD, late onset Parkinson’s disease (age at onset > 50).

^*^With df = 2, the OR value could not been given by Chi-square test.

## Discussion

In current study, we investigated the association of SNP rs150689919 and risk of PD for the first time in a cohort of Chinese subjects with PD and normal controls. However, we did not find a statistically significant difference between PD patients and controls in either allele or genotype distribution, even after stratification by gender and age at onset. Therefore, the data obtained to date do not support an association between SNP rs150689919 and sporadic PD in Chinese population.

Rare variants, which have minor allele frequencies (MAFs) <1%, might exert large effect sizes on the complex diseases. The glucocerebrosidase gene (*GBA*) serves as an example in PD: carriers of mutations in *GBA* causing Gaucher’s disease are at significantly increased risk for developing PD, with an earlier age of onset compared with PD patients who do not carry these mutations [11-13]. The identification of such variants will allow for a more complete understanding of the etiology of PD. Exome sequencing, which can screen the variants located in exons precisely and efficiently, could be used in complementation with GWAS to study the genetic basis of complex diseases. Herein, we attempted a new approach to detect susceptibility factors for sporadic PD. The DNA sequence variant rs150689919 was extracted from the whole exome sequencing results of a group of familial patients with PD and based on its potential involvement in the pathogenesis of PD. Through the combination of exome sequencing and the classical “candidate gene approach”, we can not only extended genetics studies of PD to include rare coding variants but also overcome the limitation of sample size and expensive costs associated with case–control allelic association studies built on whole-exomes.

There are several possible reasons for the negative results obtained in this study. First, the ethnic or geographic origin of subjects may account for the specific SNP frequencies responsible for PD susceptibility [14]. For instance, data from the 1000 Genomes project originated from the genomes of approximately 2500 unidentified people from ~25 populations around the world, while the subjects recruited in this study originated mainly from the mid-south of China. This might explain the difference in SNP frequencies between our controls and the 1000 Genomes project (allele T = 4.9% vs. 1.1%, respectively). Thus, the SNP with evidence suggesting an association with PD should be investigated in other independent samples. Second, when selecting candidate variants of complex disorders from exome sequences, it requires moderate sample size, or informative pedigrees to simplify the filtering of variants [15]. In this study, due to the limited sample size of the PD pedigrees and the rare frequencies of the risk alleles in the population, we failed to identify the risk variant for PD. Therefore, when we conduct exome-sequencing studies, various factors should be taken into consideration, such as the characteristics of the study population, genetic heterogeneity of the phenotype, and the sample size.

Although positive results were not obtained in our study, the combination of exome sequencing and the classical “candidate gene approach” is still useful for identifying susceptibility genes. In addition, larger sample studies should be conducted to further evaluate the potential association between SNP rs150689919 and PD or other neurodegenerative disorders in samples with different ethnic or geographic origins.

## Conclusion

To our knowledge, this is the first study to assess the frequency of rs150689919 in a cohort of Chinese PD patients and controls. Our results suggest that rs150689919 in *TET1* may not be associated with PD in ethnic Han Chinese population. However, due to the limited data in the present study, replication studies in larger sample and other populations are required.

## Abbreviations

PD: Parkinson’s disease; TET1: Tet methyl cytosine dioxygenase 1 gene; SNCA: Alpha-synuclein gene; PARK16: Parkinson disease 16; EOPD: Early onset Parkinson’s disease; LOPD: Late Onset Parkinson’s disease; PCR: Polymerase chain reaction; df: degree of freedom; OR: Odds ratio; GWAS: Genome-wide association studies.

## Competing interests

The authors declare that they have no competing interests.

## Authors’ contributions

XXL performed the genotyping, statistical analysis, and drafted the manuscript. ZZX, YYL, KL and JLW contributed to the collection of data, participated in the study design, and drafted the manuscript. JFG, XXY, KX and BST made substantial contributions to study design and cases recruitment. LS contributed to study design, molecular genetic studies, interpretation of data, and has given final approval of the version to be published. All authors read and approved the final manuscript.

## Pre-publication history

The pre-publication history for this paper can be accessed here:

http://www.biomedcentral.com/1471-2377/13/196/prepub

## Supplementary Material

Additional file 1: Table S1 Candidate variants from exome sequencing of eight PD families. Click here for file

Additional file 2: Figure S1 Pedigrees with variant *TET1* rs150689919. Each proband was indicated by an arrow. Patient II:2 in Family M17306, patient II:2 in Family M8302 and patient II:3 in Family M13742 shared the variant *TET1* rs1506899. Click here for file
