# Supplementary material for: Association study between SNP rs150689919 in the DNA demethylation gene, TET1, and Parkinson’s disease in Chinese Han population
Source: BMC Neurol. 2013 Dec 11;13:196. doi: 10.1186/1471-2377-13-196 (PMC4028872; doi:10.1186/1471-2377-13-196)
Supplement: Additional file 1: Table S1 — Candidate variants from exome sequencing of eight PD families. [file 1471-2377-13-196-S1.docx]

**Supplement Table 1.**

Candidate variants from exome sequencing of eight PD families.

| SNP ID | Gene | Alleles^a^ | Amino acid change | dbSNP135 | Reported in 1000 genomes | SIFT^b^ | PolyPhen^c^ | Cases/PD families^d^ | Hom/Het^e^ | Segregated with the disease status |
| --- | --- | --- | --- | --- | --- | --- | --- | --- | --- | --- |
| rs150689919 | *TET1* | C/**T** | S487L | Yes | Yes | 0 | probably damaging | 3/3 | Het | No |
| [rs200128812](http://browser.1000genomes.org/Homo_sapiens/Variation/Mappings?db=core;g=ENSG00000187017;r=1:6484848-6521430;v=rs200128812;vf=54789438;source=dbSNP) | *ESPN* | T/**C** | S170P | Yes | Yes | 0 | possibly damaging | 3/3 | Het | No |
| [rs199807296](http://browser.1000genomes.org/Homo_sapiens/Variation/Mappings?db=core;g=ENSG00000089639;r=19:19740285-19754476;v=rs199807296;vf=54478103;source=dbSNP) | *GMIP* | C/**G** | R838P | Yes | Yes | 0.01 | probably damaging | 3/3 | Het | No |
| rs200909431 | *TBX21* | A/**C** | T180P | Yes | Yes | 0.01 | possibly damaging | 3/3 | Het | No |
| [rs201537562](http://browser.1000genomes.org/Homo_sapiens/Variation/Mappings?db=core;g=ENSG00000169758;r=15:76352178-76521462;v=rs201537562;vf=56190242;source=dbSNP) | *C15orf27* | T/**G** | V93G | Yes | Yes | 0 | probably damaging | 3/3 | Het | No |
| r[s73840338](http://browser.1000genomes.org/Homo_sapiens/Variation/Mappings?db=core;g=ENSG00000172969;r=3:75713481-75716371;v=rs73840338;vf=17923491;source=dbSNP) | *FRG2C* | C/**A** | L202M | Yes | Yes | 0.02 | probably damaging | 3/3 | Het | No |
| [rs199759392](http://browser.1000genomes.org/Homo_sapiens/Variation/Mappings?db=core;g=ENSG00000188152;r=9:99690592-99704572;v=rs199759392;vf=54431750;source=dbSNP) | *FAM22G* | C/**G** | S137C | Yes | Yes | 0.01 | possibly damaging | 3/3 | Het | No |
| [rs139010818](http://browser.1000genomes.org/Homo_sapiens/Variation/Mappings?db=core;g=ENSG00000107593;r=10:102047903-102090243;v=rs139010818;vf=31005596;source=dbSNP) | *PKD2L1* | A/**C** | V401G | Yes | Yes | 0 | probably damaging | 3/3 | Het | No |
| [rs146069990](http://browser.1000genomes.org/Homo_sapiens/Variation/Mappings?db=core;g=ENSG00000107593;r=10:102047903-102090243;v=rs146069990;vf=37035154;source=dbSNP) | *PKD2L1* | G/**A** | S249L | Yes | Yes | 0 | possibly damaging | 3/3 | Het | No |
| [rs199920556](http://browser.1000genomes.org/Homo_sapiens/Variation/Mappings?db=core;g=ENSG00000107593;r=10:102047903-102090243;v=rs199920556;vf=54591099;source=dbSNP) | *PKD2L1* | C/**A** | D729Y | Yes | Yes | 0 | possibly damaging | 3/3 | Het | No |
| [rs201624272](http://browser.1000genomes.org/Homo_sapiens/Variation/Mappings?db=core;g=ENSG00000154639;r=21:18884700-18965897;v=rs201624272;vf=56275060;source=dbSNP) | *CXADR* | C/**G** | L250V | Yes | Yes | 0 | possibly damaging | 3/3 | Het | No |
| [rs201902185](http://browser.1000genomes.org/Homo_sapiens/Variation/Mappings?db=core;g=ENSG00000228049;r=7:102178366-102312182;v=rs201902185;vf=56549683;source=dbSNP) | *POLR2J2* | G/**A** | P78L | Yes | Yes | 0.06 | possibly damaging | 3/3 | Het | No |
| [rs201559724](http://browser.1000genomes.org/Homo_sapiens/Variation/Mappings?db=core;g=ENSG00000124557;r=6:26501449-26510650;t=ENST00000244513;v=rs201559724;vf=56211221;source=dbSNP) | *BTN1A1* | G/**C** | G80R | Yes | Yes | 0.08 | possibly damaging | 3/3 | Het | No |
| [rs142760061](http://browser.1000genomes.org/Homo_sapiens/Variation/Mappings?db=core;g=ENSG00000103056;r=16:68392231-68482591;v=rs142760061;vf=34213008;source=dbSNP) | *SMPD3* | C/**T** | G635S | Yes | Yes | 0.02 | probably damaging | 3/3 | Het | No |
| [rs144595307](http://browser.1000genomes.org/Homo_sapiens/Variation/Mappings?db=core;g=ENSG00000196507;r=X:102862379-102884618;v=rs144595307;vf=35778903;source=dbSNP) | *TCEAL3* | C/**T** | P175S | Yes | Yes | 0 | probably damaging | 3/3 | Het | No |
| [rs201465227](http://browser.1000genomes.org/Homo_sapiens/Variation/Mappings?db=core;g=ENSG00000180370;r=3:196466728-196559518;v=rs201465227;vf=56114876;source=dbSNP) | *PAK2* | G/**C** | Q101H | Yes | Yes | 0.01 | possibly damaging | 3/3 | Het | No |
| rs201386396 | *SLIT3* | T/**G** | T1142P | Yes | Yes | 0.09 | possibly damaging | 3/2 | Het | No |
| rs200979099 | *HS6ST1* | G/**T** | D87E | Yes | Yes | 0 | probably damaging | 3/2 | Het | No |
| [rs202045718](http://browser.1000genomes.org/Homo_sapiens/Variation/Mappings?db=core;g=ENSG00000094975;r=1:172501489-172580971;v=rs202045718;vf=56696635;source=dbSNP) | *C1orf9* | C/**G** | R799G | Yes | Yes | 0.01 | possibly damaging | 3/2 | Het | No |
| [rs201112792](http://browser.1000genomes.org/Homo_sapiens/Variation/Mappings?db=core;g=ENSG00000164818;r=7:766338-829190;v=rs201112792;vf=55771406;source=dbSNP) | *HEATR2* | T/**C** | L468P | Yes | Yes | 0 | probably damaging | 3/2 | Het | No |
| [rs200397471](http://browser.1000genomes.org/Homo_sapiens/Variation/Mappings?db=core;g=ENSG00000144712;r=3:12837971-12913415;v=rs200397471;vf=55065549;source=dbSNP) | *CAND2* | G/**C** | L193F | Yes | Yes | 0 | probably damaging | 3/2 | Het | No |
| [rs79546825](http://browser.1000genomes.org/Homo_sapiens/Variation/Mappings?db=core;g=ENSG00000103429;r=16:14726672-14763093;v=rs79546825;vf=23348493;source=dbSNP) | *BFAR* | A/**G** | Y38C | Yes | Yes | 0.02 | possibly damaging | 3/2 | Het | No |
| [rs146162152](http://browser.1000genomes.org/Homo_sapiens/Variation/Mappings?db=core;g=ENSG00000103429;r=16:14726672-14763093;v=rs146162152;vf=37117805;source=dbSNP) | *BFAR* | G/**A** | V148M | Yes | Yes | 0 | possibly damaging | 3/2 | Het | No |
| [rs201160764](http://browser.1000genomes.org/Homo_sapiens/Variation/Mappings?db=core;g=ENSG00000135414;r=12:56137064-56150911;v=rs201160764;vf=55818698;source=dbSNP) | *GDF11* | G/**C** | R249P | Yes | Yes | 0.01 | possibly damaging | 3/2 | Het | No |
| [rs142339148](http://browser.1000genomes.org/Homo_sapiens/Variation/Mappings?db=core;g=ENSG00000066923;r=7:99775186-99819111;v=rs142339148;vf=33854346;source=dbSNP) | *STAG3* | G/**T** | G81V | Yes | Yes | 0.01 | probably damaging | 3/2 | Het | No |
| [rs180875716](http://browser.1000genomes.org/Homo_sapiens/Variation/Mappings?db=core;g=ENSG00000149308;r=11:108027942-108093369;v=rs180875716;vf=41729794;source=dbSNP) | *NPAT* | T/**A** | S1386C | Yes | Yes | 0.04 | possibly damaging | 3/2 | Het | No |
| [rs41280175](http://browser.1000genomes.org/Homo_sapiens/Variation/Mappings?db=core;g=ENSG00000119471;r=9:115142217-115234690;v=rs41280175;vf=12517996;source=dbSNP) | *HSDL2* | A/**T** | K91I | Yes | Yes | 0.01 | probably damaging | 3/2 | Het | No |
| [rs186016030](http://browser.1000genomes.org/Homo_sapiens/Variation/Mappings?db=core;g=ENSG00000183624;r=3:128997671-129025029;v=rs186016030;vf=46877262;source=dbSNP) | *C3orf37* | C/**G** | L112V | Yes | Yes | 0.01 | probably damaging | 3/2 | Het | No |

^a^Risk allele in bold.

^b^Effects of SNP predicted by SIFT: Ranges from 0 to 1. The amino acid substitution is predicted damaging if the score is <=0.05, and tolerated if the score is >0.05.

^c^ Effects of SNP predicted by PolyPhen: Probably damaging, i.e., it is with high confidence supposed to affect protein function or structure; Possibly damaging, i.e., it is supposed to affect protein function or structure; Benign, most likely lacking any phenotypic effect;. Unknown, when in some rare cases, the lack of data do not allow PolyPhen to make a prediction

^d^Number of cases and families with variants at the position.

^e^Het/Hom: “Het” is short for “heterozygous”; “Hom” is short for “homozygous”.
