# Supplementary material for: Association study between SNP rs150689919 in the DNA demethylation gene, TET1, and Parkinson’s disease in Chinese Han population
Source: BMC Neurol. 2013 Dec 11;13:196. doi: 10.1186/1471-2377-13-196 (PMC4028872; doi:10.1186/1471-2377-13-196)
Supplement: Additional file 2: Figure S1 — Pedigrees with variant TET1 rs150689919. Each proband was indicated by an arrow. Patient II:2 in Family M17306, patient II:2 in Family M8302 and patient II:3 in Family M13742 shared the variant TET1 rs1506899. [file 1471-2377-13-196-S2.docx]

**
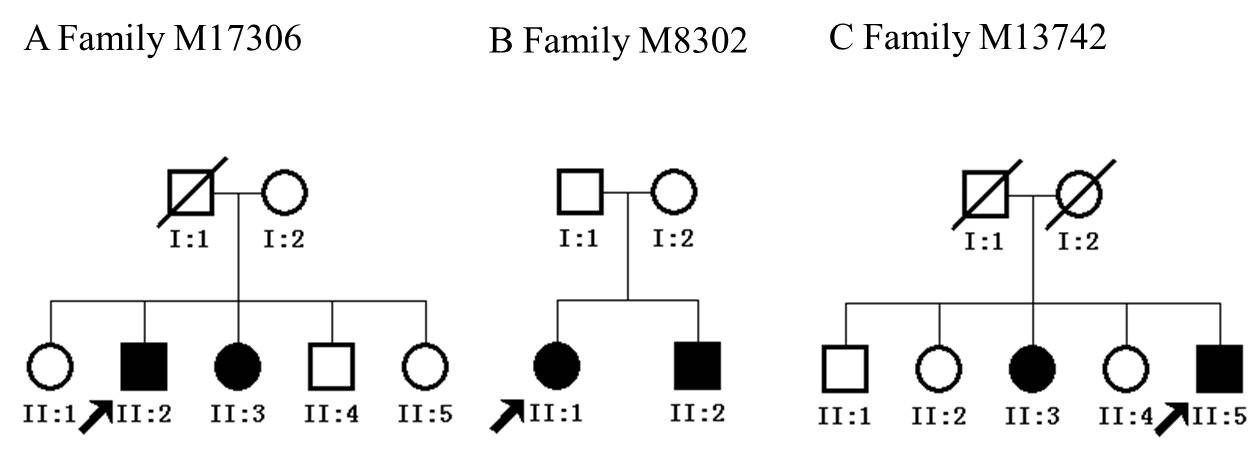
**

**Supplement Figure 1.** Pedigrees with variant *TET1* rs150689919. Each proband was indicated by an arrow. Patient Ⅱ:2 in Family M17306, patient Ⅱ:2 in Family M8302 and patient Ⅱ:3 in Family M13742 shared the variant *TET1* rs1506899
